# Supplementary material for: Genome-wide CRISPR screening identifies new regulators of glycoprotein secretion
Source: Wellcome Open Res. 2020 Jan 16;4:119. Originally published 2019 Aug 9. [Version 2] doi: 10.12688/wellcomeopenres.15232.2 (PMC6979480; doi:10.12688/wellcomeopenres.15232.2)
Supplement: Peer review history

# Peer review: Reviewer response for version 2

**DOI:** 10.21956/wellcomeopenres.17182.r37605

**Published:** 2020-01-23

**Authors:** Hesso Farhan

**Affiliations:** 1 Institute of Basic Medical Sciences, Department of Molecular Medicine, University of Oslo, Oslo, Norway

I have no further comments or suggestion.

I confirm that I have read this submission and believe that I have an appropriate level of expertise to confirm that it is of an acceptable scientific standard.

# Peer review: Reviewer response for version 2

**DOI:** 10.21956/wellcomeopenres.17182.r37604

**Published:** 2020-01-21

**Authors:** David Stephens

**Affiliations:** 1 School of Biochemistry, Faculty of Life Sciences, University of Bristol, Bristol, UK

Some minor text changes have been made but further experiments don't address key concerns e.g. around the effects on glycoprotein versus general protein secretion.

I confirm that I have read this submission and believe that I have an appropriate level of expertise to confirm that it is of an acceptable scientific standard.

# Peer review: Reviewer response for version 1

**DOI:** 10.21956/wellcomeopenres.16623.r36252

**Published:** 2019-08-28

**Authors:** David Stephens

**Affiliations:** 1 School of Biochemistry, Faculty of Life Sciences, University of Bristol, Bristol, UK

This is an interesting manuscript that applies CRISPR gene editing approaches to screen for components of the glycoprotein secretion machinery. It appears to be carefully conducted with appropriate analysis applied to the screen. I am not an expert in that area and so cannot comment on the design, execution, or analysis of the screen further. 

 The results are clearly presented and some key "hits" defined. I would have liked to have read more about the overlap between the current work and previous efforts e.g. whole genome RNAi screens in mammalian cells and flies ^1^^,^^2^^,^^3^. Particularly given the previous use of ssHRP as a reporter. 

 Fundamentally the screen is really testing those genes required for localization of galectin-3 to the cell surface, this is not quite the same as a global analysis of glycoprotein secretion. Perhaps validation with other lectins, or of specific glycoproteins, or even an unbiased glycoproteomics approach might have been appropriate - at least for the "hits" that were worked up. Really, I would have liked to see some analysis of other endogenous glycoproteins as well as non-glycosylated secretory proteins. In particular, it is not clear to me whether the effects seen are indeed general or specific to galectin-3.

 The analysis of five of the key "hits" is quite preliminary but the work is carefully conducted and nicely presented. This provides some framework for others to build on these data. The link between defects in glycoprotein secretion and Golgi organization are not really explored and this could have been built on to provide more insight. 

 A fundamental limitation for me is that none of the validation work has been done in any system other than HeLa cells. Given the literature on the role of GPR161 in cilia ^4^, one might have hoped to see some work-up done in ciliated cells. 

 I expect that this will stand alone as a useful data resource and can certainly be built on by others.

 Specific comments: 

- RNAi efficacy (important for Figure 5) is not validated anywhere that I could see. 
- The colocalization analysis is limited. GolginA5 and TGN46 should not overlap significantly as they localize to distinct parts of the Golgi/TGN (5D), therefore I have doubts over the robustness of this readout. In Figure 5C colocalization of GPR161 and GolginA5 does not appear robust to me and is not quantified. The images shown are low resolution and more detailed analysis is warranted. Nocodazole treatment of cells to scatter the stacked Golgi would be useful here. 
- The integrity of the microtubule network should be checked as disruption of this could result in altered Golgi morphology (but not likely to secretion per se). The two phenotypes might be unconnected. 
- The conclusion that GPR161 might recruit GolginA5 is premature. The data do not provide sufficient support for this. 

I confirm that I have read this submission and believe that I have an appropriate level of expertise to confirm that it is of an acceptable scientific standard, however I have significant reservations, as outlined above.

## References

[1] : Functional genomics reveals genes involved in protein secretion and Golgi organization. *Nature*.2006;439(7076) : 10.1038/nature04377 604-7 10.1038/nature04377 16452979

[2] : Genome-wide RNAi screening identifies human proteins with a regulatory function in the early secretory pathway. *Nat Cell Biol*.2012;14(7) : 10.1038/ncb2510 764-74 10.1038/ncb2510 22660414

[3] : A genome-wide RNA interference screen identifies two novel components of the metazoan secretory pathway. *EMBO J*.2010;29(2) : 10.1038/emboj.2009.350 304-14 10.1038/emboj.2009.350 19942856PMC2824459

[4] : The ciliary G-protein-coupled receptor Gpr161 negatively regulates the Sonic hedgehog pathway via cAMP signaling. *Cell*.2013;152(1-2) : 10.1016/j.cell.2012.12.026 210-23 10.1016/j.cell.2012.12.026 23332756

# Peer review: Reviewer response for version 1

**DOI:** 10.21956/wellcomeopenres.16623.r36185

**Published:** 2019-08-27

**Authors:** Hesso Farhan

**Affiliations:** 1 Institute of Basic Medical Sciences, Department of Molecular Medicine, University of Oslo, Oslo, Norway

The work by Popa and colleagues from the Moreau group reports on the identification of new regulators of glycoprotein secretion using a CRISPR screen.

 The manuscript is written well and is easy to follow. Most of the data are of strong quality and therefore, I am supportive of the indexing of this work. I have a few concerns that could be easily addressed by a revision.

1. The title implies that the paper identifies regulators of glycoprotein secretion. How sure are the authors that these new proteins regulate glycoprotein trafficking, and not general trafficking? If they want to maintain the prefix glyco-, then they should show that non-glycosylated proteins such as albumin are not affected.
2. The authors use the term “Golgi fragmentation”, but this is not what I see. In many cases, the Golgi is simply aberrant in shape (stretched, or extended). Is it possible to revisit the data to show quantification of Golgi “fragments”? I think it is safer to use the term “aberrant Golgi structure”. The authors could use the Golgi compactness index developed originally by Fred Bard (2003).
3. It is hard to appreciate the effects on ERES, with the magnification and image quality the authors use. In my experience, a 20X magnification does not permit reliable judging of ERES. The authors should do better ERES imaging and quantification for the 2-3 most important hits. Especially for TMEM220, which they consider a regulator of ER-Golgi trafficking. This needs to be experimentally supported.
4. The authors state/conclude that GRP161 serves to recruit GolginA5 to the Golgi. I don’t think that the data support this claim. A similar perturbation of GolgiA5 staining is seen with TMEM220. Could these not be simply a reflection of a distorted Golgi structure?
5. General comment: the work does not compare the results to previous screens by Rainer Pepperkok ^1^, Hesso Farhan ^2^ and Fred Bard ^3^. I am not sure why the authors chose to ignore this previous work, despite its obvious relevance to the current paper. Not only should these three papers be cited, but a comparison of the hits should be made.

I confirm that I have read this submission and believe that I have an appropriate level of expertise to confirm that it is of an acceptable scientific standard, however I have significant reservations, as outlined above.

## References

[1] : Genome-wide RNAi screening identifies human proteins with a regulatory function in the early secretory pathway. *Nat Cell Biol*.2012;14(7) : 10.1038/ncb2510 764-74 10.1038/ncb2510 22660414

[2] : MAPK signaling to the early secretory pathway revealed by kinase/phosphatase functional screening. *J Cell Biol*.2010;189(6) : 10.1083/jcb.200912082 997-1011 10.1083/jcb.200912082 20548102PMC2886346

[3] : RNAi screening reveals a large signaling network controlling the Golgi apparatus in human cells. *Mol Syst Biol*.2012;8: 10.1038/msb.2012.59 629 10.1038/msb.2012.59 23212246PMC3542528
